# Effects of *Panax ginseng* on Tumor Necrosis Factor-α-Mediated Inflammation: A Mini-Review

**DOI:** 10.3390/molecules16042802

**Published:** 2011-03-30

**Authors:** Davy CW Lee, Allan SY Lau

**Affiliations:** 1 Cytokine Biology Group, Department of Paediatrics and Adolescent Medicine, The University of Hong Kong, Pokfulam, Hong Kong SAR, China; 2 Molecular Chinese Medicine Laboratory, Li Ka Shing Faculty of Medicine, The University of Hong Kong, Pokfulam, Hong Kong SAR, China

**Keywords:** *Panax ginseng*, ginsenosides, cytokines, immunomodulation, inflammation

## Abstract

*Panax ginseng* is one of the most commonly used Chinese medicines in China, Asia and Western countries. The beneficial effects of ginseng have been attributed to the biological activities of its constituents, the ginsenosides. In this review, we summarize recent publications on the anti-inflammatory effects of ginseng extracts and ginsenosides on cellular responses triggered by different inducers including endotoxin, tumor necrosis factor-alpha (TNF-α), interferon-gamma and other stimuli. Proinflammatory cytokines, chemokines, adhesion molecules and mediators of inflammation including inducible nitric oxide synthase, cyclooxygenase-2 and nitric oxide orchestrate the inflammatory response. Ginseng extracts and ginsenosides including Rb_1_, Rd, Rg_1_, Rg_3_, Rh_1_, Rh_2_, Rh_3 _and Rp_1_ have been reported to have anti-inflammatory properties in different studies related to inflammation. Ginsenosides inhibit different inducers-activated signaling protein kinases and transcription factor nuclear factor-kappaB leading to decreases in the production of cytokines and mediators of inflammation. The therapeutic potential of ginseng on TNF-α-mediated inflammatory diseases is also discussed. Taken together, this summary provides evidences for the anti-inflammatory effects of ginseng extracts and ginsenosides as well as the underlying mechanisms of their effects on inflammatory diseases.

## 1. Introduction

*Panax ginseng* C.A. Meyer (ginseng) was first classified by German botanist Nees Von Esenbeck in 1833. It was later renamed by Russian botanist Carl Anton Meyer in 1843 [1]. The name of the genus *Panax* was derived from a Greek word which literally means all-healing or panacea. [2]. Ginseng has been used as an herbal remedy in ancient China, Korea, Japan and the Far East for more than 5,000 years and the medical efficacy of ginseng was documented in ancient Asian literatures [1,3]. Following studies of the uses of ginseng in humans, the beneficial effects of ginseng were recognized in Western countries by the 18th century [4].

Ginseng belongs to the family *Araliaceae*. Currently, twelve to thirteen species of ginseng have been identified in the genus *Panax* and their geographical distributions are summarized in a recent review [1,3]. Three major species of ginseng including *Panax ginseng* C. A. Meyer, *Panax quinquefolium* L and *Panax notoginseng* have been extensively investigated for their physiological and pharmacological effects on the human body [5,6,7].

## 2. Active Components of *Panax ginseng*

Ginseng species contain multiple active constituents including ginsenosides, polysaccharides, peptides, phytosterols, polyacetylenes, polyacetylenic alcohols and fatty acids that have been shown to have different effects on carbohydrate and lipid metabolism, cognition and angiogenesis as well as on the function of neuroendocrine, immune, cardiovascular and central nervous systems [2,4].

The ginsenosides are the major biologically active compounds of ginseng. Recent studies have shown that ginsenosides play important roles in the pharmacological effects of ginseng [3]. Structurally, ginsenosides belong to the triterpene dammarane-type saponins and comprise triterpenoidal glycosides of the dammar type with glucose, arabinose, xylose or rhamnose [8]. Over 30 different ginsenosides have been isolated from ginseng and identified [3]. Twenty-two subtypes of ginsenosides including Ra_1_ Ra_2_, Ra_3_ Rb_1_, Rb_2_, Rb_3_, Rc, Rd, Rg_3_ and Rh_2 _belong to the 20(*S*)-protopanaxadiol group, whereas eleven subtypes of ginsenosides including Re, Rf, Rg_1_, Rg_2 _and Rh_1 _belong to the 20(*S*)-protopanaxatriol group. Additionally, the oleanolic acid group contains ginsenoside Ro [2,9]. However, the concentration and bioactivities of ginsenosides in different ginsengs could vary by up to 20% [3]. The variability is related to the species, the part of the plant, the period of cultivation, and the processing method used [9,10]. Different pharmacological effects of ginsenosides have been reported and different combination of ginsenosides including 20(*S*)-protopanaxadiol and 20(*S*)-protopanaxatriols have been used in treating inflammatory diseases [11]. Moreover, ginseng contains other compounds including phenolic compounds, polyacetylenes, ginsenoyne, sesquiterpenes, methoxypyrazine, alkylpyrazine derivatives, sesquiterpene alcohols, panasinsanols, β-caboline, and neutral or acidic polysaccharides [8,10].

## 3. Inflammation and Cytokines

Inflammation is a complex biological response of our body to harmful external stimuli including microbial infections and chemical toxins. The inflammatory response is characterized by several steps including coordinate activation of signaling pathways, expression of proinflammatory cytokines, chemokines and adhesion molecules in resident tissue cells, as well as infiltration of leukocytes mainly macrophages, neutrophils and dendritic cells and mediators of inflammation from the vascular system to remove the harmful stimuli and to initiate the healing process [12,13].

Leukocytes or endothelial cells produce proinflammatory or anti-inflammatory cytokines depending on their activities on regulating inflammation. Proinflammatory cytokines including tumor necrosis factor-α (TNF-α) and interleukin-1β (IL-1β) have been shown to play central roles in the pathogenesis of both acute and chronic inflammatory diseases [14,15]. Other proinflammatory cytokines include IL-17, IL-18 and the newly identified member of IL-1 family, IL-33, whereas anti-inflammatory cytokines including IL-10 and transforming growth factor-β negatively regulate or dampen the over-activated inflammatory responses [16,17,18,19,20,21,22,23,24,25].

TNF-α plays critical roles in the initiation and amplification of inflammatory response [26,27,28,29]. TNF-α signaling pathways are triggered by binding of TNF-α to one of the two distinct cell surface receptors, TNF-R1 and TNF-R2. TNF-R1 mediates largely the proinflammatory and apoptotic pathways activated by TNF-α, whereas TNF-R2 is associated with TNF-mediated tissue repair and angiogenesis. Following binding of TNF-α to TNF-R1, downstream signaling pathways are triggered by the recruitment of cytosolic adaptor proteins including receptor interacting protein, TNFR associated factor2, and FAS-associated death domain through specific protein-protein interaction domains. The protein signaling complexes recruit distinct enzymes resulting in the activation of different signaling pathways including caspases, the transcription factor nuclear factor-kappaB (NF-κB) and mitogen activated protein kinases (MAPK), and eventually induced transcription of inflammatory mediators [28].

## 4. Anti-Inflammatory Effects of Ginseng Extracts and Ginsenosides

Ginseng extracts and the ginsenosides have been shown a wide range of beneficial effects against human diseases and their potential therapeutic effects have been attributed to its immunomodulatory, anti-oxidant and anti-inflammatory activities [30]. The anti-inflammatory effects of ginseng extracts and ginsenosides are associated with their properties of cytokine regulation and phagocytosis in innate immunity, as well as activation of T- and B- lymphocytes [31,32,33,34,35,36]. Moreover, reports have shown their adjuvant activities for different kinds of vaccines [9,37]. Previous reports of anti-inflammatory effects of ginseng extracts and ginsenosides will be described in following sections.

### 4.1. Panax ginseng Extract

Ginsan, the acid polysaccharide extract from ginseng, has been shown to have immunomodulatory effects in various reports. Ginsan inhibits the production of TNF-α, IL-1β, IL-6, IL-12, IL-18 and interferon-gamma (IFN-γ), and enhances the phagocytic activity of macrophages in *Staphylococcus aureus*-infected mice [38,39]. In addition, ginsan inhibits the activation of MAPK pathways including p38MAPK and c-Jun N-terminal kinases (JNK), and NF-κB in the infected mice [38]. Furthermore, ginsan is shown to have anti-asthmatic effects including reduction of airway hyperresponsiveness, and decreases of eosinophils in ovalbumin-treated mice. These effects are equivalent to those of dexamethasone treatment and could be partially mediated by enhancing the synthesis of cyclooxygenases and prostaglandin (PGE)_2_ [40].

Ginsan exerts protective effects on mice with carbon tetrachloride-induced liver injury via inhibition of carbon tetrachloride-induced cytochrome P450 pathways and lipid peroxidation. In addition, ginsan attenuates the production of proinflammatory cytokines including IL-1β, IFN-γ and chemokines including monocyte chemoattractant protein (MCP)-1/CCL-2, macrophage inflammatory protein (MIP)-2β/CXCL-2 and KC/CXCL-1 leading to reduce leukocyte infiltration and inflammatory response [41]. However, ginsan stimulates the expression of IL-12, TNF-α and Major Histocompatibility Complex (MHC) class II molecules in murine dendritic cells and the proliferation of allogeneic CD4(+) T lymphocytes [42].

### 4.2. Panax Notoginseng Extracts

The anti-atherosclerotic and anti-arthritic effects of *Panax notoginseng* saponins (PNS) in rodents have been reported recently. PNS show anti-atherosclerotic effects on apolipoprotein E-deficient mice. The levels of serum lipid, IL-6 and TNF-α in PNS-treated mice are lower than those of control mice. The anti-atherosclerotic effects of PNS could be due to the inhibition of TNF-α induced monocyte adhesion and the expression of endothelial adhesion molecules including vascular cell adhesion molecule-1 (VCAM-1) and intercellular cell adhesion molecule-1 (ICAM-1) [7]. In addition, PNS is shown to attenuate the pathological changes of atherosclerosis induced by zymosan A in rat. The expression levels of integrins, IL-18, IL-1β, matrix metalloproteinase-2 (MMP-2), MMP-9, and NF-κB are decreased in PNS-treated rats [6]. On the other hand, PNS attenuates the degree of hepatic fibrosis in mice treated with carbon tetrachloride. The levels of serum transforming growth factor-β1, TNF-α and IL-6 decrease in PNS-treated mice compared with the control mice whereas the level of IL-10 increases, suggesting that PNS has certain therapeutic effects on hepatic fibrosis by regulating the imbalance of pro-fibrotic and anti-fibrotic cytokines [43]. BT-201, n-butanol extract of *Panax notoginseng*, has been shown the anti-arthritic effects on collagen-induced arthritis (CIA) mice [5]. BT-201 also decreases the production of TNF-α, IL-1β, inducible nitric oxide, and MMP-13, possibly via the suppression of NF-κB, ERK, p38 MAPK and JNK activation [5]. Moreover, the ethanol extract of *Panax notoginseng* roots inhibits the expressions of TNF-α, IL-6 and CD40 in DC2.4 murine dendritic cells stimulated by Toll-like receptor ligands including lipopolysachharide (LPS), CpG and/or polyriboinosinic-polyribocytidylic acid [poly(I:C)]. However, the underlying mechanisms of the anti-inflammatory effects of *Panax notoginseng* extracts remains investigated [44].

### 4.3. Panax Quinquefolius Extracts

The ethanol extract of *Panax quinquefolius* selectively inhibits the expression of iNOS in RAW264.7 cells induced by LPS by suppressing the Signal Transducers and Activators of Transcription protein (STAT)/iNOS signaling pathways [45]. Another report shows that the ethanol extracts of *Panax quinquefolius* can suppress the expression of inducible nitric oxide synthase (iNOS), cyclooxygenase-2 (COX-2) and p53 in mice with experimental colitis. The mucosal and DNA damage associated with colitis in these mice are also suppressed by the *Panax quinquefolius* extract [46].

### 4.4. Rb_1_

Ginsenoside Rb_1_ inhibits the over-expression of VCAM-1 and over-production of superoxide anion in TNF-α treated endothelial cells by suppressing MAPK and NF-κB signaling pathways [47]. Rb_1_ also inhibits the productions of inflammatory mediators including IL-8 and PGE₂ in HaCaT human keratinocytes induced by capsaicin [48]. Rb_1_ inhibits capsaicin-induced calcium influx and NF-κB activity through capsaicin receptor also known as transient receptor potential channel vanilloid subtype 1 signaling pathways [48].

The anti-arthritic effect of Rb_1_ has been reported in collagen-induced arthritis CIA mice. Rb_1_ inhibits TNF-α induction in peripheral blood mononuclear cells, fibroblast-like synoviocytes, and chondrocytes stimulated by IFN-γ, LPS or IL-1β. In addition, Rb1 suppresses the severity of inflammatory response in CIA mice by reducing TNF-α expression, cartilage destruction and cell infiltration in the arthritic joints of the mice [49].

### 4.5. Rd

Ginsenoside Rd has been shown neuroprotective effects on a rat model of transient focal cerebral ischemia. Rd reduces the accumulation of the early detected oxidative DNA, protein and lipid peroxidation products in rats subjected to transient middle cerebral artery occlusion. Rd suppresses the inflammatory responses by inhibiting expressions of iNOS and COX-2 and microglial activation [50].

### 4.6. Rg_1_

Ginsenoside Rg_1_ inhibits the expression of TNF-α, iNOS and ionized calcium binding adaptor molecule-1 in both cerebral cortex and hippocampus in mice intracerebroventricular injected of LPS by suppressing NF-κB and MAPK pathways [51]. Consistent report shows that Rg_1_ suppresses NF-κB pathways leading to decrease production of TNF-α and nitric oxide in N9 microglial cells induced by LPS. Rg_1_ can be a potent inhibitor of microglial cells activation-mediated neuro-degenerative diseases [52]. On the other hand, Rg_1_ induces Th1 dominant cytokines production such as IFN-γ and IL-2 in CD4(+) T cells of mice infected with *Candida albicans*. Such activities help the host to resist the risk of dissemination of candidiasis [53].

### 4.7. Rg_3_

Ginsenoside Rg_3_ inhibits the COX-2 expression as well as NF-κB and activator protein-1 activations in 12-O-tetradecanoylphorbol-13-acetate-induced mouse skin and human promyelocytic leukemia cells [54]. Rg_3 _also reduces TNF-α, IL-1β, IL-6, MCP-1, MIP-1γ expressions in BV-2 murine microglial cells treated with beta-amyloid, indicating that Rg_3_ prevents neurotoxicity by inhibiting proinflammatory cytokines production in microglial cells [55]. Rg_3_ may have anti-atherosclerotic activities in the vasculature. Rg_3_ suppresses the expressions of TNF-α induced VCAM-1 and ICAM-1 in ECV 304 human endothelial cells and prevents the endothelial cells undergo apoptosis through the Akt-mediated, caspases-activated pathways [56,57].

### 4.8. Rh_1_

Ginsenoside Rh_1_ may provide beneficial effects on neuroinflammatory diseases by modulating the microglial activation. Rh_1_ suppresses production of nitric oxide, reactive oxygen species and TNF-α in BV2 microglial cells induced by IFN-γ. The study for iNOS promoter shows that IFN-γ-activated JAK/STAT and ERK signaling pathways, and their downstream transcription factors including NF-κB, interferon regulatory factor-1 and STAT1 was inhibited by Rh_1_ [58]. In addition, Rh_1_ induces expressions of IL-10 and hemeoxygenase-1 expression via activation of cAMP-dependent protein kinase signaling pathways. On the other hand, Rh_1_ inhibits activation of NF-κB and extracellular signal regulated kinase (ERK1/2) signaling pathways and expressions of iNOS and COX-2 in LPS-stimulated microglia [59]. Hence, the anti-inflammatory effects of Rh_1_ alleviate the severity of neuro-degenerative diseases.

A recent report shows that Rh_1_ can alleviate inflammatory symptoms in oxazolone-induced atopic dermatitis-like skin lesions in hairless mice by suppressing the level of IgE and IL-6 in peripheral blood [60]. Additionally, Rh_1_ reduces histamine release in rat peritoneal mast cells and the IgE-mediated passive cutaneous anaphylaxis reaction in mice [61]. The anti-allergic effects of Rh_1_ may due to anti-inflammatory activities since Rh_1_ inhibits the expression of iNOS and COX-2 in LPS-induced RAW264.7 murine macrophages by preventing phosphorylation and degradation of the inhibitor of NF-κB (I-κB) [61,62].

### 4.9. Rh_2_ and Rh_3_

Ginsenoside Rh_2_ has specific inhibitory effects on activation of NF-κB and JNK signaling pathways in human astroglial cells-induced by TNF-α [63]. In addition, Rh_2_ inhibits the DNA binding activity of activator protein 1 (AP-1) and production of nitric oxide, COX-2, TNF-α and IL-1β in BV2 cells induced by LPS and IFN-γ. On the other hand, Rh_2_ can increase the expression of IL-10 in BV2 cells [64]. Ginsenosides Rh_2_ and Rh_3_ significantly inhibit LPS-induced microglial cells activation by suppressing expression of inflammatory mediators including iNOS, MMP-9, TNF-α, and IL-1β [65].

### 4.10. Rp_1_

Ginsenoside Rp_1_ is a newly reported ginsenoside derivative that show inhibitory effects on IL-1β expression in LPS-treated RAW264.7 cells by blocking the activation of NF-κB signaling pathways [66]. Our recent report demonstrated that the ethanol extract of *Panax ginseng* inhibits expressions of TNF-α-inducible cytokines and signaling proteins in human promonocytic cells. Ginseng extract suppresses TNF-α-induced chemokine CXCL-10 expression by inactivating the ERK1/2 pathway. We also showed that the suppressive effect of ginseng extract on TNF-α induced-CXCL-10 transcription could be due to the collective effects of ginsenosides mixtures instead of a single ginsenoside [67]. The inflammatory study models, targeted regulatory proteins and effects on inflammatory mediators for ginseng extracts and ginsenosides are summarized in Table 1.

**Table 1:** Summary of anti-inflammatory effects of ginseng extracts and ginsenosides.

Ginseng extract/Ginsenoside	Experimental model	Regulatory proteins	Effects on inflammatory mediators	References
*Panax ginseng* acid polysaccharide extract	Ovalbumin-treated mice	-	↓Cyclooxygenases, PGE2	[40]
*Panax ginseng* acid polysaccharide extract	*Staphylococcus aureus-*infected mice	p38MAPK, JNK and NF-κB	↓IL-1β, IL-6, IL-12, IL-18, IFN-γ	[38,39]
*Panax ginseng* acid polysaccharide extract	Carbon tetrachloride-induced mice	-	↓CCL-2, CXCL-2, CXCL-1	[41]
*Panax ginseng* acid polysaccharide extract	Murine dendritic cells	-	↑IL-12, TNF-α, MHC-II	[42]
*Panax ginseng* ethanol extract	TNFα-induced U937 cells	ERK	↓CXCL-10	[67]
*Panax notoginseng* Saponins	Apolipoprotein-E deficient mice	-	↓IL-6, TNF-α, VCAM, ICAM	[7]
*Panax notoginseng* Saponins	Zymosan A-induced mice	NF-κB	↓MMP-2, MMP-9, IL-18, IL-1β, integrins	[6]
*Panax notoginseng* Saponins	Carbon tetrachloride-induced mice	-	↓TGF-β, TNF-α, IL-6↑IL-10	[43]
*Panax notoginseng* n-butanol extract	Collagen-induced arthritis mice	p38MAPK, JNK, ERK and NF-κB	↓TNF-α, IL-1β, iNOS, MMP-13	[5]
*Panax notoginseng* ethanol extract	LPS, CpG or poly(I:C)-induced DC2.4 murine dendritic cells	-	↓IL-6, TNF-α, CD40	[44]
*Panax quinquefolius* ethanol extract	LPS-induced RAW murine macrophages	STAT	↓iNOS	[45]
*Panax quinquefolius* ethanol extract	Experimental colitis mice	-	↓iNOS, COX-2, p53	[46]
Rb_1_	TNF-induced endothelial cells	MAPK and NF-κB	↓TNF-α	[47]
Rb_1_	Capsaicin-induced HaCaT human keratinocyte	NF-κB	↓IL-8, PGE2	[48]
Rb_1_	IFN-γ, LPS/IL-1β−induced CIA mice	-	↓TNF-α in PBMC, fibroblast-like synoviocytes and chondrocytes	[49]
Rd	Transient focal cerebral ischemia	-	↓iNOS, COX-2	[50]
Rg_1_	LPS-injected mice	p38MAPK, JNK and NF-κB	↓TNF-α, iNOS and ionized calcium binding adaptor molecule-1	[51]
Rg_1_	LPS-N9 microglial cells	NF-κB	↓TNF-α, nitric oxide	[52]
Rg_1_	CD4(+) T cells of Candida albicans-infected mice	-	↑IL-2, IFN-γ	[53]
Rg_3_	TPA-induced mouse skin cells and U937 promyelocytic leukemia cells	AP-1, NF-κB	↓COX-2	[54]
Rg_3_	Beta-amyloid-induced BV2 murine microglial cells	-	↓TNF-α, IL-1β, IL-6, MCP-1, MIP-1γ	[55]
Rg_3_	TNF-α-induced-ECV304 human endothelial cells	AKT	↓VCAM-1, ICAM-1	[56,57]
Rh_1_	IFN-γ-BV2 murine microglial cells	ERK, STAT1, IRF-1 and NF-κB	↓Nitric oxide, reactive oxygen species, TNF-α	[58]
Rh_1_	LPS-stimulated microglia	cAMP-dependent protein kinase	↑IL-10, hemeoxygenase-1	[59]
Rh1	LPS-stimulated microglia	ERK and NF-κB	↓iNOS, COX-2	[59]
Rh_1_	Oxazolone-induced atopic dermatitis skin lesion in mice	-	↓IL-6, IgE in peripheral blood↑Foxp3	[60]
Rh_1_	LPS-induced RAW murine macrophages	NF-κB	↓iNOS, COX-2	[61,62]
Rh_2_	TNF-α-induced human astroglial cells	JNK and NF-κB	-	[63]
Rh_2_	LPS/IFN-γ-induced BV2 microglial cells	AP-1	↓Nitric oxide, COX-2, TNF-α, IL-1β	[64]
Rh_2_	Rh2-induced BV2 murine microglial cells	-	↓IL-10	[64]
Rh_2_ & Rh_3_	LPS-induced microglial cells	-	↓iNOS, MMP-9, IL-1β,TNF-α	[65]
Rp_1_	LPS-induced RAW murine macrophages	NF-κB	↓IL-1β	[66]

AP-1: activator protein 1; COX: cyclooxygenase; ERK: extracellular signal regulated kinase; ICAM: intercellular cell adhesion molecule; iNOS: inducible nitric oxide synthase; JNK: c-Jun N-terminal kinases; LPS: lipopolysaccharide; MAPK: mitogen activated protein kinases; MCP: monocyte chemoattractant protein; MHC: major histocompatibility complex; MIP: macrophage inflammatory protein; MMP: matrix metalloproteinase; NF-kB: nuclear factor-kappaB; PGE: prostaglandin; PBMC: peripheral blood mononuclear cells; STAT: Signal Transducers and Activators of Transcription protein; TGF: Transforming growth factor; TNF: tumor necrosis factor; TPA: 12-*O*-tetradecanoylphorbol-13-acetate; VCAM: vascular cell adhesion molecule; ↓: downregulation of expression; ↑: upregulation of expression.

## 5. Application of Ginseng and Ginsenoside to TNF-α Mediated Inflammatory Diseases

TNF-α is a pleiotropic cytokine and its biological functions are varied and complex. It interacts with an array of other cytokines to initiate a cascade of cellular activities downstream. TNF-α induces expressions of vascular adhesion molecules on the endothelial cells and chemokines on macrophages and neutrophils to enhance cell trafficking [68,69,70,71,72]. In addition, TNF-α stimulates phagocytic functions of monocytes and macrophages to clear invading pathogens or cell debris. Hence, TNF-α production in acute infection is mostly beneficial to the host. However, excess uncontrolled production of TNF-α can cause over-induction of cytokines and chemokines including IL-1, IL-6, IL-8, growth-regulated oncogene, CXCL-10 and CCL-5. Additionally, the release of glucocorticoids may enhance local inflammation and cause tissue damage [14,73]. Several autoimmune and inflammatory diseases including rheumatoid arthritis [72], septic shock [74], Crohn’s disease, psoriasis [75], and inflammatory bowel disease [76,77] are associated with dysregulated TNF-α production. As TNF-α is a good therapeutic target for treating inflammatory diseases, the identification of TNF-α antagonists from medicinal herbs and natural products, for example ginseng, could definitely provide an alternative for the treatment of debilitating inflammatory diseases. However, it has to emphasize that the anti-inflammatory effects of ginseng and/or ginsenosides cannot be attributed to lowering TNF-α only but also their immunoregulatory properties on innate and adaptive immunity as mentioned earlier.

## 6. Conclusions

Ginseng contains a complex mixture of chemical constituents that have multiple and diverse physiological effects on the human body. The anti-atherosclerotic, anti-arthritic, anti-oxidative and anti-allergic effects of ginseng and the ginsenosides in *in vitro* and *in vivo* studies may due to the their anti-inflammatory activity. However, the detailed molecular mechanisms of the anti-inflammatory activities of ginseng extracts and ginsenosides remain to be investigated.

During the last few decades, bioactivity-guided approach have been widely applied for isolation or identification of the bioactive compounds from herbs [78]. This approach can be extended further to another level by combining the different bioassay and the rapidly developed liquid chromatography and tandem mass spectrometry, and liquid chromatography and nuclear magnetic resonance technologies to determine not only the native bioactive compounds in medicinal herbs but also their structural interactions to the targets at the molecular level [79].

## References

[1] Yun T.K. (2001). Brief introduction of *Panax ginseng* C.A. Meyer. J. Korean Med. Sci..

[2] Gillis C.N. (1997). *Panax ginseng* pharmacology: A nitric oxide link?. Biochem. Pharmacol..

[3] Yue P.Y., Mak N.K., Cheng Y.K., Leung K.W., Ng T.B., Fan D.T., Yeung H.W., Wong R.N. (2007). Pharmacogenomics and the Yin/Yang actions of ginseng: anti-tumor, angiomodulating and steroid-like activities of ginsenosides. Chin. Med..

[4] Attele A.S., Wu J.A., Yuan C.S. (1999). Ginseng pharmacology: Multiple constituents and multiple actions. Biochem. Pharmacol..

[5] Chang S.H., Choi Y., Park J.A., Jung D.S., Shin J., Yang J.H., Ko S.Y., Kim S.W., Kim J.K. (2007). Anti-inflammatory effects of BT-201, an *n*-butanol extract of *Panax notoginseng*, observed *in vitro* and in a collagen-induced arthritis model. Clin. Nutr..

[6] Zhang Y.G., Zhang H.G., Zhang G.Y., Fan J.S., Li X.H., Liu Y.H., Li S.H., Lian X.M., Tang Z. (2008). *Panax notoginseng* saponins attenuate atherosclerosis in rats by regulating the blood lipid profile and an anti-inflammatory action. Clin. Exp. Pharmacol. Physiol..

[7] Wan J.B., Lee S.M., Wang J.D., Wang N., He C.W., Wang Y.T., Kang J.X. (2009). *Panax notoginseng* reduces atherosclerotic lesions in ApoE-deficient mice and inhibits TNF-alpha-induced endothelial adhesion molecule expression and monocyte adhesion. J. Agric. Food Chem..

[8] Yun T.K. (1999). Update from Asia. Asian studies on cancer chemoprevention. Ann. NY Acad. Sci..

[9] Jia L., Zhao Y. (2009). Current evaluation of the millennium phytomedicine--ginseng (I): Etymology, pharmacognosy, phytochemistry, market and regulations. Curr. Med. Chem..

[10] Lee M., Sorn S., Baek S., Jang S., Kim S. (2009). Antioxidant and apoptotic effects of korean white ginseng extracted with the same ratio of protopanaxadiol and protopanaxatriol saponins in human hepatoma HepG2 cells. Ann. NY Acad. Sci..

[11] Nah S.Y., Park H.J., McCleskey E.W. (1995). A trace component of ginseng that inhibits Ca2+ channels through a pertussis toxin-sensitive G protein. Proc. Natl. Acad. Sci. USA.

[12] Sacca R., Cuff C.A., Ruddle N.H. (1997). Mediators of inflammation. Curr. Opin. Immunol..

[13] Dinarello C.A. (2010). Anti-inflammatory agents: Present and future. Cell.

[14] Bradley J.R. (2008). TNF-mediated inflammatory disease. J. Pathol..

[15] Clark I.A. (2007). How TNF was recognized as a key mechanism of disease. Cytokine Growth Factor Rev..

[16] Dinarello C.A. (2000). Proinflammatory cytokines. Chest.

[17] Opal S.M., DePalo V.A. (2000). Anti-inflammatory cytokines. Chest.

[18] Carriere V., Roussel L., Ortega N., Lacorre D.A., Americh L., Aguilar L., Bouche G., Girard J.P. (2007). IL-33, the IL-1-like cytokine ligand for ST2 receptor, is a chromatin-associated nuclear factor *in vivo*. Proc. Natl. Acad. Sci. USA.

[19] Dinarello C.A. (2010). IL-1: Discoveries, controversies and future directions. Eur. J. Immunol..

[20] Latz E. (2010). The inflammasomes: mechanisms of activation and function. Curr. Opin. Immunol..

[21] Onishi R.M., Gaffen S.L. (2010). Interleukin-17 and its target genes: Mechanisms of interleukin-17 function in disease. Immunology.

[22] Pappu R., Ramirez-Carrozzi V., Ota N., Ouyang W., Hu Y. (2010). The IL-17 family cytokines in immunity and disease. J. Clin. Immunol..

[23] Liew F.Y., Pitman N.I., McInnes I.B. (2010). Disease-associated functions of IL-33: the new kid in the IL-1 family. Nat. Rev. Immunol..

[24] Mosser D.M., Zhang X. (2008). Interleukin-10: New perspectives on an old cytokine. Immunol. Rev..

[25] Kim E.Y., Moudgil K.D. (2008). Regulation of autoimmune inflammation by pro-inflammatory cytokines. Immunol. Lett..

[26] Park K.M., Bowers W.J. (2010). Tumor necrosis factor-alpha mediated signaling in neuronal homeostasis and dysfunction. Cell Signal.

[27] Hehlgans T., Pfeffer K. (2005). The intriguing biology of the tumour necrosis factor/tumour necrosis factor receptor superfamily: players, rules and the games. Immunology.

[28] Chen G., Goeddel D.V. (2002). TNF-R1 signaling: a beautiful pathway. Science.

[29] Aggarwal B.B. (2003). Signalling pathways of the TNF superfamily: A double-edged sword. Nat. Rev. Immunol..

[30] Radad K., Gille G., Liu L., Rausch W.D. (2006). Use of ginseng in medicine with emphasis on neurodegenerative disorders. J. Pharmacol. Sci..

[31] Ho L.J., Juan T.Y., Chao P., Wu W.L., Chang D.M., Chang S.Y., Lai J.H. (2004). Plant alkaloid tetrandrine downregulates IkappaBalpha kinases-IkappaBalpha-NF-kappaB signaling pathway in human peripheral blood T cell. Br. J. Pharmacol..

[32] Mizuno M., Yamada J., Terai H., Kozukue N., Lee Y.S., Tsuchida H. (1994). Differences in immunomodulating effects between wild and cultured *Panax ginseng*. Biochem. Biophys. Res. Commun..

[33] Nakaya T.A., Kita M., Kuriyama H., Iwakura Y., Imanishi J. (2004). *Panax ginseng* induces production of proinflammatory cytokines via toll-like receptor. J. Interferon Cytokine Res..

[34] Shin J.Y., Song J.Y., Yun Y.S., Yang H.O., Rhee D.K., Pyo S. (2002). Immunostimulating effects of acidic polysaccharides extract of *Panax ginseng* on macrophage function. Immunopharmacol. Immunotoxicol..

[35] Sonoda Y., Kasahara T., Mukaida N., Shimizu N., Tomoda M., Takeda T. (1998). Stimulation of interleukin-8 production by acidic polysaccharides from the root of *Panax ginseng*. Immunopharmacology.

[36] Tan B.K., Vanitha J. (2004). Immunomodulatory and antimicrobial effects of some traditional chinese medicinal herbs: A review. Curr. Med. Chem..

[37] Song X., Hu S. (2009). Adjuvant activities of saponins from traditional Chinese medicinal herbs. Vaccine.

[38] Ahn J.Y., Choi I.S., Shim J.Y., Yun E.K., Yun Y.S., Jeong G., Song J.Y. (2006). The immunomodulator ginsan induces resistance to experimental sepsis by inhibiting Toll-like receptor-mediated inflammatory signals. Eur. J. Immunol..

[39] Ahn J.Y., Song J.Y., Yun Y.S., Jeong G., Choi I.S. (2006). Protection of *Staphylococcus aureus*-infected septic mice by suppression of early acute inflammation and enhanced antimicrobial activity by ginsan. FEMS Immunol. Med. Microbiol..

[40] Lim Y.J., Na H.S., Yun Y.S., Choi I.S., Oh J.S., Rhee J.H., Cho B.H., Lee H.C. (2009). Suppressive effects of ginsan on the development of allergic reaction in murine asthmatic model. Int. Arch. Allergy Immunol..

[41] Shim J.Y., Kim M.H., Kim H.D., Ahn J.Y., Yun Y.S., Song J.Y. (2010). Protective action of the immunomodulator ginsan against carbon tetrachloride-induced liver injury via control of oxidative stress and the inflammatory response. Toxicol. Appl. Pharmacol..

[42] Kim M.H., Byon Y.Y., Ko E.J., Song J.Y., Yun Y.S., Shin T., Joo H.G. (2009). Immunomodulatory activity of ginsan, a polysaccharide of *Panax ginseng*, on dendritic cell. Korean J. Physiol. Pharmacol..

[43] Peng X.D., Dai L.L., Huang C.Q., He C.M., Yang B., Chen L.J. (2009). Relationship between anti-fibrotic effect of *Panax notoginseng* saponins and serum cytokines in rat hepatic fibrosis. Biochem. Biophys. Res. Commun..

[44] Rhule A., Rase B., Smith J.R., Shepherd D.M. (2008). Toll-like receptor ligand-induced activation of murine DC2.4 cells is attenuated by *Panax notoginseng*. J. Ethnopharmacol..

[45] Ichikawa T., Li J., Nagarkatti P., Nagarkatti M., Hofseth L.J., Windust A., Cui T. (2009). American ginseng preferentially suppresses STAT/iNOS signaling in activated macrophages. J. Ethnopharmacol..

[46] Jin Y., Kotakadi V.S., Ying L., Hofseth A.B., Cui X., Wood P.A., Windust A., Matesic L.E., Pena E.A., Chiuzan C., Singh N.P., Nagarkatti M., Nagarkatti P.S., Wargovich M.J., Hofseth L.J. (2008). American ginseng suppresses inflammation and DNA damage associated with mouse colitis. Carcinogenesis.

[47] Chai H., Wang Q., Huang L., Xie T., Fu Y. (2008). Ginsenoside Rb1 inhibits tumor necrosis factor-alpha-induced vascular cell adhesion molecule-1 expression in human endothelial cells. Biol. Pharm. Bull..

[48] Huang J., Qiu L., Ding L., Wang S., Wang J., Zhu Q., Song F., Hu J. (2010). Ginsenoside Rb1 and paeoniflorin inhibit transient receptor potential vanilloid-1-activated IL-8 and PGE production in a human keratinocyte cell line HaCaT. Int. Immunopharmacol..

[49] Kim H.A., Kim S., Chang S.H., Hwang H.J., Choi Y.N. (2007). Anti-arthritic effect of ginsenoside Rb1 on collagen induced arthritis in mice. Int. Immunopharmacol..

[50] Ye R., Yang Q., Kong X., Han J., Zhang X., Zhang Y., Li P., Liu J., Shi M., Xiong L., Zhao G. (2010). Ginsenoside Rd attenuates early oxidative damage and sequential inflammatory response after transient focal ischemia in rats. Neurochem. Int..

[51] Hu J.F., Song X.Y., Chu S.F., Chen J., Ji H.J., Chen X.Y., Yuan Y.H., Han N., Zhang J.T., Chen N.H. (2011). Inhibitory effect of ginsenoside Rg1 on lipopolysaccharide-induced microglial activation in mice. Brain Res..

[52] Wu C.F., Bi X.L., Yang J.Y., Zhan J.Y., Dong Y.X., Wang J.H., Wang J.M., Zhang R., Li X. (2007). Differential effects of ginsenosides on NO and TNF-alpha production by LPS-activated N9 microglia. Int. Immunopharmacol..

[53] Lee J. H., Han Y. (2006). Ginsenoside Rg1 helps mice resist to disseminated candidiasis by Th1 type differentiation of CD4+ T cell. Int. Immunopharmacol..

[54] Keum Y.S., Han S.S., Chun K.S., Park K.K., Park J.H., Lee S.K., Surh Y.J. (2003). Inhibitory effects of the ginsenoside Rg3 on phorbol ester-induced cyclooxygenase-2 expression, NF-kappaB activation and tumor promotion. Mutat. Res..

[55] Joo S.S., Yoo Y.M., Ahn B.W., Nam S.Y., Kim Y.B., Hwang K.W., Lee do I. (2008). Prevention of inflammation-mediated neurotoxicity by Rg3 and its role in microglial activation. Biol. Pharm. Bull..

[56] Min J.K., Kim J.H., Cho Y.L., Maeng Y.S., Lee S.J., Pyun B.J., Kim Y.M., Park J.H., Kwon Y.G. (2006). 20(*S*)-Ginsenoside Rg3 prevents endothelial cell apoptosis via inhibition of a mitochondrial caspase pathway. Biochem. Biophys. Res. Commun..

[57] Hien T.T., Kim N.D., Kim H.S., Kang K.W. (2010). Ginsenoside Rg3 inhibits tumor necrosis factor-alpha-induced expression of cell adhesion molecules in human endothelial cells. Pharmazie.

[58] Jung J.S., Kim D.H., Kim H.S. (2010). Ginsenoside Rh1 suppresses inducible nitric oxide synthase gene expression in IFN-gamma-stimulated microglia via modulation of JAK/STAT and ERK signaling pathways. Biochem. Biophys. Res. Commun..

[59] Jung J.S., Shin J.A., Park E.M., Lee J.E., Kang Y.S., Min S.W., Kim D.H., Hyun J.W., Shin C.Y., Kim H.S. (2010). Anti-inflammatory mechanism of ginsenoside Rh1 in lipopolysaccharide-stimulated microglia: critical role of the protein kinase A pathway and hemeoxygenase-1 expression. J. Neurochem..

[60] Zheng H., Jeong Y., Song J., Ji G.E. (2011). Oral administration of ginsenoside Rh1 inhibits the development of atopic dermatitis-like skin lesions induced by oxazolone in hairless mice. Int. Immunopharmacol..

[61] Park E.K., Choo M.K., Han M.J., Kim D.H. (2004). Ginsenoside Rh1 possesses antiallergic and anti-inflammatory activities. Int. Arch. Allergy Immunol..

[62] Oh G.S., Pae H.O., Choi B.M., Seo E.A., Kim D.H., Shin M.K., Kim J.D., Kim J.B., Chung H.T. (2004). 20(*S*)-Protopanaxatriol, one of ginsenoside metabolites, inhibits inducible nitric oxide synthase and cyclooxygenase-2 expressions through inactivation of nuclear factor-kappaB in RAW 26.7 macrophages stimulated with lipopolysaccharide. Cancer Lett..

[63] Choi K., Kim M., Ryu J., Choi C. (2007). Ginsenosides compound K and Rh(2) inhibit tumor necrosis factor-alpha-induced activation of the NF-kappaB and JNK pathways in human astroglial cells. Neurosci. Lett..

[64] Bae E.A., Kim E.J., Park J. S., Kim H. S., Ryu J. H., Kim D. H. (2006). Ginsenosides Rg3 and Rh2 inhibit the activation of AP-1 and protein kinase A pathway in lipopolysaccharide/interferon-gamma-stimulated BV-2 microglial cells. Planta Med..

[65] Park J.S., Park E.M., Kim D.H., Jung K., Jung J.S., Lee E.J., Hyun J.W., Kang J.L., Kim H.S. (2009). Anti-inflammatory mechanism of ginseng saponins in activated microglia. J. Neuroimmunol..

[66] Kim B.H., Lee Y.G., Park T.Y., Kim H.B., Rhee M.H., Cho J.Y. (2009). Ginsenoside Rp1, a ginsenoside derivative, blocks lipopolysaccharide-induced interleukin-1beta production via suppression of the NF-kappaB pathway. Planta Med..

[67] Lee D.C., Yang C.L., Chik S.C., Li J.C., Rong J.H., Chan G.C., Lau A.S. (2009). Bioactivity-guided identification and cell signaling technology to delineate the immunomodulatory effects of *Panax ginseng* on human promonocytic U937 cells. J. Transl. Med..

[68] Barbara J.A., Van ostade X., Lopez A. (1996). Tumour necrosis factor-alpha (TNF-alpha): the good, the bad and potentially very effective. Immunol. Cell Biol..

[69] Pfeffer K., Matsuyama T., Kundig T.M., Wakeham A., Kishihara K., Shahinian A., Wiegmann K., Ohashi P.S., Kronke M., Mak T.W. (1993). Mice deficient for the 55 kd tumor necrosis factor receptor are resistant to endotoxic shock, yet succumb to L. monocytogenes infection. Cell.

[70] Rothe J., Lesslauer W., Lotscher H., Lang Y., Koebel P., Kontgen F., Althage A., Zinkernagel R., Steinmetz M., Bluethmann H. (1993). Mice lacking the tumour necrosis factor receptor 1 are resistant to TNF-mediated toxicity but highly susceptible to infection by *Listeria monocytogenes*. Nature.

[71] Pasparakis M., Kousteni S., Peschon J., Kollias G. (2000). Tumor necrosis factor and the p55TNF receptor are required for optimal development of the marginal sinus and for migration of follicular dendritic cell precursors into splenic follicles. Cell. Immunol..

[72] Flynn J.L., Goldstein M.M., Chan J., Triebold K.J., Pfeffer K., Lowenstein C.J., Schreiber R., Mak T.W., Bloom B.R. (1995). Tumor necrosis factor-alpha is required in the protective immune response against *Mycobacterium tuberculosis* in mice. Immunity.

[73] Elenkov I.J., Chrousos G.P. (2002). Stress hormones, proinflammatory and antiinflammatory cytokines, and autoimmunity. Ann. NY Acad. Sci..

[74] Beutler B., Cerami A. (1989). The biology of cachectin/TNF--a primary mediator of the host response. Annu. Rev. Immunol..

[75] Atzeni F., Turiel M., Capsoni F., Doria A., Meroni P., Sarzi-Puttini P. (2005). Autoimmunity and anti-TNF-alpha agents. Ann. NY Acad. Sci..

[76] Kassiotis G., Kollias G. (2001). Uncoupling the proinflammatory from the immunosuppressive properties of tumor necrosis factor (TNF) at the p55 TNF receptor level: implications for pathogenesis and therapy of autoimmune demyelination. J. Exp. Med..

[77] Kollias G., Douni E., Kassiotis G., Kontoyiannis D. (1999). On the role of tumor necrosis factor and receptors in models of multiorgan failure, rheumatoid arthritis, multiple sclerosis and inflammatory bowel disease. Immunol. Rev..

[78] Queiroz E.F., Wolfender J.L., Hostettmann K. (2009). Modern approaches in the search for new lead antiparasitic compounds from higher plants. Curr. Drug Targets.

[79] Jiang Y., David B., Tu P., Barbin Y. (2010). Recent analytical approaches in quality control of traditional Chinese medicines—A review. Anal. Chim. Acta.

